# Pet ownership and survival of European older adults

**DOI:** 10.1007/s10433-022-00739-6

**Published:** 2022-11-04

**Authors:** Konstantinos Christopoulos, Vasiliki Benetou, Elena Riza, Nikos Pantazis

**Affiliations:** Department of Hygiene, Epidemiology and Medical Statistics, Medical School, National and Kapodistrian University of Athens, Mikras Asias 75, Athens, 115 27 Greece

**Keywords:** Companion animals, Human–animal interaction, Mortality, Pet exposure, Survival analysis

## Abstract

With pet ownership on the rise, millions of individuals are exposed to this environmental exposure. Although the subject has been largely studied, more evidence is needed to clarify the potential association of pet ownership with human health. The aim of this research is to study the potential association of pet exposure (any pet, cat, dog, bird, fish) with all-cause, cardiovascular and cancer mortality of older ($\ge$ 50 years) European residents. To this end, a total of 23,274 participants from the Survey of Health Ageing and Retirement in Europe (SHARE) were employed (median follow-up 119 months). All-cause mortality (5163 events), as well as cardiovascular (CVD) (1832 events), and cancer mortality (1346 events) were examined using Cox Proportional Hazards models for their relation with pet exposure at baseline. Stratified analyses were also performed by gender and for single or multi-person households. No significant association was observed for any of the pets with all-cause mortality on the whole sample and the fully adjusted models. In stratified analyses, bird exposure significantly increased the risk of all-cause mortality in women [Hazard Ratio $(\text {HR})=1.23$; 95% CI 1.04–1.44] as well as women living alone $(\text {HR}=1.38$; 95% CI 1.02–1.85). Cause-specific models revealed an increased risk of death for women bird owners for causes other than cancer and CVD $(\text {HR}=1.40$; 95% CI 1.05–1.99). In conclusion, bird ownership may be negatively associated with survival of older women in Europe.

## Introduction

People and animals have coexisted for thousands of years. In past times, their relationship revolved around daily work and husbandry, but in recent years, companionship has been one of the main purposes for ownership. Worldwide, the numbers of household pets have been increasing (GfK 2016). In Europe, rough estimates suggest that 38% of European households own at least one pet, with cats being the most prevalent (in numbers) followed by dogs, birds and other mammalian, aquatic, and reptilian animals (FEDIAF 2020).

This trend has led several researchers to study the potentially beneficial effect of pet exposure on the health and longevity of their owners. As a result, a plethora of observational studies on physical (Curl et al. 2016; Mičková et al. 2019; Parslow et al. 2005; Raina et al. 1999; Taniguchi et al. 2018) or mental health (Colombo et al. 2006; Enmarker et al. 2015; Friedmann et al. 2011; Garrity et al. 1989; Parslow et al. 2005; Powell et al. 2019; Raina et al. 1999; Taniguchi et al. 2018), with exposures spanning from in utero (Xu et al. 2020) and early life (Casas et al. 2013), to late years (Scheibeck et al. 2011), make up the literature.

The mental and physical health of older individuals has been a focal point in human–animal health research. Recent studies claim that pets, and dogs in particular, can be beneficial for socially isolated older adults during the COVID-19 pandemic (Ikeuchi et al. 2021) and might even reduce the frailty incidence risk of their walkers during periods of population mobility-limiting mandates (e.g. lockdown measures) (Taniguchi et al. 2019). Hughes et al. (2020) provide a systematic review which highlights the beneficial effect in various mental health outcomes as well as evidence for improved CVD risk factors, amongst pet owners.

On the other hand, researchers have also identified potential hazards associated with pet ownership such as allergies (Hölscher et al. 2002), zoonotic diseases (Grant and Olsen 1999), the increased risk of falls in older individuals (Kurrle et al. 2004), and bite injuries with or without subsequent infection (Feldman et al. 2004). However, the majority of the existing research is cross sectional; hence, robust evidence is limited. Flegr and Preiss (2019) also make a case for eliminating the volunteer bias which is present in many studies as the self-selection of pet owners can also lead to measurement error of their self-perceived health status when they are informed about the aim of the study.

A more robust methodology—compared to cross-sectional designs—with a clear time dimension is the time-to-event (TTE) analysis. In the case of pets, even with a TTE analysis, several difficulties lie in the measurement of the exposure and interpretation of the results. Nevertheless, all-cause, non-established CVD^1^ and cancer mortality have been studied with regard to pet exposure. The studies concern predominantly US or northern European populations; hence, their generalisability is limited.

Several survival studies have shown no association of dog or cat exposure with all-cause mortality (Ding et al. 2018; Qureshi et al. 2009; Torske et al. 2017; Gillum and Obisesan 2010). Mubanga et al. (2017) provides the only study where all-cause mortality is inversely associated with dog ownership, although no lifestyle controls were included in the analysis. The evidence is limited for a protective association against CVD mortality in dog owners. In two large sample cohort studies, Ding et al. (2018) in England found no association, while Mubanga et al. (2017) in Sweden found a strong protective effect ($\text {HR}=0.64$). Interestingly enough, Qureshi et al. (2009) argue that *past* cat ownership is inversely associated with CVD and Myocardial Infraction (MI) deaths. Ogechi et al. (2016) also found a very protective association against CVD and stroke deaths in current (at baseline) cat owners. Because the number of events in these last studies was very limited, further investigation is required.

Cancer mortality has also been the subject of investigation, but only in US population samples. Despite the fact that no association was found in the cancer incidence of US women owners by Garcia et al. (2016), a number of studies using the National Health and Nutrition Examination Survey (NHANES) III data were performed. Cat or bird ownership was associated with lung cancer in women (Adhikari et al. 2019), and additionally, cat exposure was also linked to increased risk of colorectal cancer (Adhikari et al. 2020). Buck et al. (2020) examining all-cancer mortality also found an increased risk for women owners of cats or birds, probably a result of the hazards described in the two previous cancer-specific studies. 

This study aims to explore whether pet exposure (any pet, dog, cat, bird, fish, as well as interactions between pets) assessed at baseline is associated with survival among older European residents. To this end, we model all-cause, CVD, and cancer mortality using data from the SHARE project (Börsch-Supan et al. 2013). In an effort to shed light into the matter, our study aims to contribute to the literature by pooling information from large samples from a diverse set of European countries. Previous studies with large samples were at national level; hence, external validity was limited. Moreover, we use a large set of cross-country comparable control variables and additionally test for unobserved heterogeneity.

## Materials and methods

### The SHARE project

The SHARE project is an ongoing longitudinal household survey of European residents which started in 2004 and is performed approximately every 2 years. It focuses primarily on individuals over the age of 50 but younger participants are also included in case of cohabitation. The survey uses a probability sample, although the sampling framework varies between countries.^2^ The data are collected via computer-assisted personal interviews (CAPI) and participation rates, although highly variable between countries, were approximately^3^ 60% and 50% for household and individual, respectively (Bergmann et al. 2017). Additionally, a self-completion drop-off questionnaire containing more sensitive questions is distributed to participants. The response rate for this was 81% on average for the first wave (Bergmann et al. 2017).The validity, reliability and final translation of the questionnaire were pre-tested in a probability subsample.

All the data for this analysis were extracted from the SHARE databases of waves 1 through 8. According to the methodology book (Börsch-Supan and Jürges 2005) ‘SHARE underwent a thorough review of ethical standards by the University of Mannheim’s internal review board (IRB)’. SHARE has obtained all the necessary consents of the individuals whose personal data were obtained.

### Study participants

**Fig. 1:**
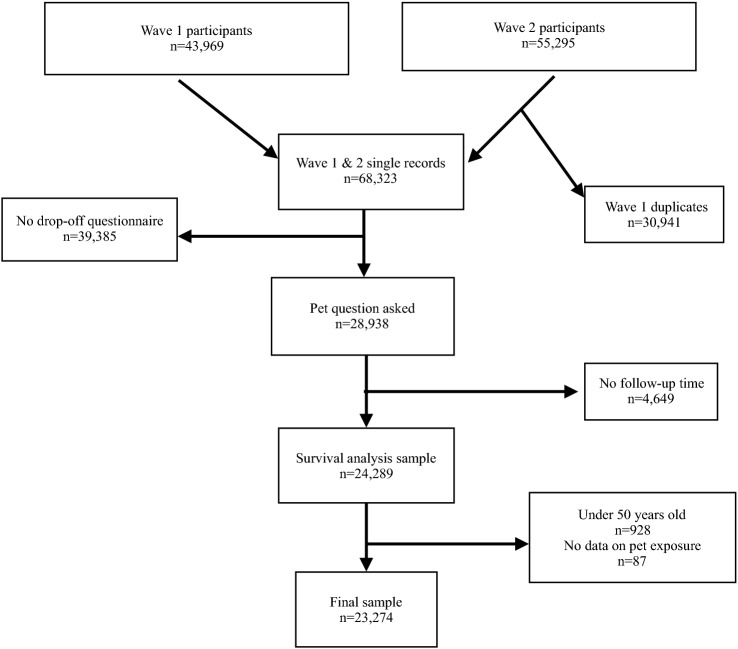
Flowchart for the selection of study participants

Since data on pet ownership and its components were available only in waves 1 and 2 from the drop-off questionnaires, participants who did not complete them were excluded from the study. Individuals without follow-up were also excluded, as were those under the age of 50 and those without any pet exposure information. Our final sample consists of 23,274 individuals from 15 countries,^4^ each country representing from 9.9 to 2.2% of the sample. Despite the exclusion criteria, the sample maintains the basic characteristics of the SHARE sample regarding the age and gender distribution. Figure 1 provides a schematic of the sample formation and inclusion criteria.

### Survival data

The date of birth and entry in study were recorded at baseline. Exit from the study occurred in the case of death or due to right censoring. In case of death, all relevant information about the date and cause of death were provided by a proxy-respondent (e.g. family or household member, neighbour, friend, etc.). The right censoring time point was the last interview date of the participant. The reported causes of death were re-recoded by the authors into cancer, CVD^5^ and Other.^6^

### Pet exposure

Pet ownership data and its components were extracted from the baseline supplementary written questionnaire. The question regarding ownership was ‘Do you currently have one or more of the following pets in your household?’, with dog, cat, bird, fish, other pets and no pets in household, as available responses. The overall pet ownership dummy variable was created using information from the aforementioned questions, taking the value 1 if one of the 5 first responses was selected, and zero otherwise.

### Covariates

Our analysis includes a large set of covariates including, demographic, socioeconomic, lifestyle, and medical, in an effort to control for all possible confounders. It is worth mentioning that the answers are self-reported and all covariates were measured at baseline, unless stated otherwise. Refusing, not knowing, and not remembering to answer was decoded as a missing value.

#### Identifying potential confounders

The literature on the determinants of pet ownership is very limited in number, especially, with regard to generalisability. Therefore, it provides no clear evidence as to what may confound the relationship between pet exposure and survival. Nevertheless, there is some evidence that points toward education, social class, gender and age (Eller et al. 2008; Westgarth et al. 2010). Eller et al. (2008) also provide evidence for confounding by various allergic conditions. Sharpley et al. (2020) additionally show that depressed individuals had increased odds of owning a dog. Other proposed factors such as household size could also play a role, although the link with survival is nebulous. Westgarth et al. (2010) propose correctly that each pet should be modelled individually; however, in the absence of studies, especially for less popular pets, the task is rather difficult. Regardless, an attempt was made for pet specific models in the less than fully adjusted specifications.

#### Demographic variables

Several demographic variables were included in the analysis such as gender (male, female); age at baseline (continuous); country of residence (see footnote 4), marital status (married and living together with spouse, registered partnership, married living separated from spouse, never married, divorced, widowed); been in a nursing home^7^ (temporarily, permanently, no); and finally, the first wave of appearance in SHARE (wave 1 or wave 2). It is worth mentioning that age at baseline serves additionally as a control for the birth cohort effects, not explicitly modelled in our study.

#### Socioeconomic variables

Key socioeconomic variables including household size^8^ (integer $>0$) and employment status (retired, employed or self-employed, unemployed, permanently sick or disabled, homemaker, other) were included. The main interest of course lies in the measurement of education and wealth. For the former, education was measured and harmonised using the 1997 International Standard Classification of Education (ISCED-97) by SHARE, and then recoded into 7 ordinal categories ranging from no education to PhD education, which were used in our analysis. Finally, the question ‘Thinking of your household’s total monthly income, would you say that your household is able to make ends meet?’ (with great difficulty, with some difficulty, fairly easily, easily) was used as a proxy for income/wealth. This variable ensures comparability between participants from different countries since it captures the purchasing power as well as the financial struggle at the household level.

#### Lifestyle variables

Several behavioural risk factors such as tobacco and alcohol consumption, physical inactivity and the Body Mass Index (BMI) were also included in the list of potential confounders. Metric BMI was calculated from the self-reported weight and height or the respondents as kg/m$^2$ by SHARE and was included as a continuous variable in our analysis. The frequency of physical activity was measured by two variables, one accounting for moderate and one for vigorous activity^9^ (more than once a week, once a week, one to three times a month; hardly ever, or never). Tobacco consumption as current smoking status (yes, currently smoke; never smoked daily for at least one year; no, I have stopped), and alcohol consumption as frequency in the last 6 months (daily or almost every day, five or six days a week, three or four days a week, once or twice a week, once or twice a month, less than once a month, not at all in the last 3 months).

#### Medical information

This category includes several variables regarding the physical and mental health of individuals. The self-perceived health was also measured using the US scale (excellent, very good, good, fair, poor). Mental health was measured using the Euro-D depression scale.^10^ Physical health was measured by two variables, namely the number of chronic conditions and the number of symptoms. These variables are the sum of a list of conditions and symptoms, respectively. Asthma diagnosis (yes, no) was included to control for potential confounding from allergic conditions. Cancer or CVD diagnosis dummies were used only when modelling these specific hazards. Finally, disability was measured as a count of mobility, arm function and fine motor limitations, from a ten item list.

### Statistical analysis

The time scale used for the analysis was the attained age. This time scale is more appropriate for longitudinal surveys since it accounts for left-truncation (delayed entry) and groups subjects in similar risks together (Kom et al. 1997; Thiébaut and Bénichou 2004). Lamarca et al. (1998) also argue that this time scale is more appropriate for the survival analysis of older adults. Despite the data being recorded in months (monthly time interval censoring), continuous time analysis was preferred due to the large median follow-up (119 months).

Cox Proportional Hazards (PH) were used throughout the analysis, although Gompertz PH models also fitted the data very well. Ties were handled with the Breslow method. The proportionality assumption was tested for each pet exposure using scaled Schoenfeld residuals and was not rejected. Cluster robust standard errors were used throughout the analysis to account for the dependence at the household level.

Pet exposure was treated as a time-invariant variable due to data availability. A model was fitted for each pet, pet interaction as well as overall pet ownership. For each of these models, four different models were employed. Model 1 controls for basic demographic factors; Model 2 controls additionally for known confounders from the literature; Model 3 controls additionally for variables whose role in the causal mechanism remains unclear and is discussed in Sect. “Modelling and inferential difficulties”; and Model 4 (the fully adjusted model) controls for every covariate mentioned in Sect. “Covariates”. Moreover, stratified analyses were performed by gender and household size, where modification was identified in past studies (Mubanga et al. 2017).

Missing values were excluded from the analysis with pairwise deletion. Missing values on the cause of death (${n}=380$, 7.36%) were treated as censored in the cause-specific analysis at the time of death. The analyses are divided in all-cause and cause-specific mortality and were performed with STATA/ MP 13.0 (StataCorp LP, TX, US). Lastly, 0.05 was used as the level for statistical significance.

## Results

### Sample description

The mean age of the participants at baseline was 64.2 years with a standard deviation of 9.8. Women were slightly more than men (54.2%) and the average household had 2.3 persons. The average participant had at least some secondary education and only 11.5% stated that their household struggles financially. Average BMI was 26.6 and only 19% were currently smokers. Some vigorous physical activity was present in 59% of the sample, while some moderate physical activity was reported in 88%. Health-wise, the average participants had 1.6 chronic conditions, 1.7 symptoms, 1.5 mobility limitations and a 2.4 score on the Euro-D depression scale, at baseline.

Table 1 presents a comparison of descriptive statistics for some key variables between pet and non-pet owners. Non-pet owners were 3 years older on average, and they also had slightly higher education and better finances. Pet owners had slightly larger household size, worse smoking habits but better physical activity. No real differences existed in gender, BMI as well as in all the medical covariates.

**Table 1:** Selected demographic, socioeconomic, lifestyle and medical characteristics of the participants by pet ownership

		Pet owners	Non-pet owners	*p* value$\ddag$
		*N*=13,965(60.05%)	*N*=9,292 (39.95%)	
*Demographic*				
Age (at baseline)	Mean (SD)	62.17 (9.15)	65.63 (10.01)	< 0.001
Gender	Male	46.56%	45.34%	0.070
	Female	53.44%	54.66%	
Household size	1	13.22%	22.82%	< 0.001
	2	52.59%	56.30%	
	3+	34.19%	20.88%	
*Socioeconomic*				
Education (ISCED-97)$\dagger$	Median (IQR)	3 (3–1)	3 (3–1)	0.002
Financial management of household	With great difficulty	12.22%	11.06%	< 0.001
	With some difficulty	30.29%	26.64%	
	Fairly easily	34.18%	33.50%	
	Easily	23.32%	28.81%	
*Lifestyle*				
Smoking	Currently smoke	21.76%	17.21%	< 0.001
	Never smoked daily for at least one year	50.05%	54.10%	
	Have stopped	28.19%	28.69%	
BMI (kg/m$^2$)	Mean (SD)	26.74 (4.45)	26.45 (4.30)	< 0.001
Physical activity (vigorous)	More than once a week	38.44%	34.40%	< 0.001
	Once a week	13.84%	13.70%	
	One to three times a month	9.36%	9.17%	
	Hardly ever, or never	38.36%	42.72%	
*Medical*				
Depression (Euro-D)	Median (IQR)	2 (4–1)	2 (4–1)	0.021
Disability (mobility limitations count)	Median (IQR)	0 (2–0)	0 (2–0)	0.034
Symptoms (count)	Median (IQR)	1 (2–0)	1 (2–0)	0.343
Chronic conditions (count)	Median (IQR)	1 (2–0)	1 (2–1)	0.001
Asthma diagnosis	Yes	4.80%	4.78%	0.950

$\dagger$ The ISCED-97 ranges from 0 to 7 with higher numbers indicating higher education. $\ddag$ *p* values for comparisons come from: mean t-tests for continuous variables, for median comparisons from Wilcoxon rank-sum tests, for dichotomous variables from Fisher’s 2-sided exact test, and for ordinal categorical variables from Pearson $\chi ^2$ tests

### Survival description

During 231,183 person-years of follow-up a total of 5163 deaths were recorded. The mortality rate was 2.23 deaths per 100 person-years. The median survival was up to the age of 86.6 [95% CI (86.3–86.8)], while the mean follow-up was 119 months or about 12 years. For cause-specific mortality, cancer deaths were 1346, and CVD deaths were 1832, while deaths from other causes were 1605. Table 2 entails descriptive statistics for the survival of different pet owners.

**Table 2:** Median survival with 95% confidence intervals and mortality rates for different pet exposures

	Median survival (in years)	Mortality rate (per 1000 person-years)
*Any pet*	85.8 [85.2–86.3]	18.9
*No pet*	86.9 [86.6–87.2]	24.6
*Cat*	86.0 [85.1–86.8]	19.4
*Dog*	85.4 [84.8–86.1]	19.2
*Bird*	85.3 [84.3–86.3]	22.1
*Fish*	86.9 [85.0–89.6]	12.9

Median survival and mortality rates refer to all-cause mortality

### All-cause mortality

The basic model (Model 1) pointed to a negative significant association between survival and pet exposure for dog and bird. Once established confounders were introduced in Model 2 all these associations lost their statistical significance and their effects were attenuated. Further adjusting (Model 3) further moved the hazard ratio toward the null. In the fully adjusted model, little changed in terms of significance and magnitude. Moreover, none of the interactions between pets was statistically significant ($p \,\text {values} > 0.1$, results not shown). Table 3 provides the Cox PH models estimates for all-cause mortality.

**Table 3:** Cox PH all-cause mortality hazard ratios and 95% confidence intervals for pet exposures, $N=23{,}274$, $\text {Events}=5163$

	Model 1		Model 2		Model 3		Model 4	
	HR	CI	HR	CI	HR	CI	HR	CI
*Any pet*	1.09**	[1.03–1.16]	1.03	[0.97–1.10]	1.02	[0.96–1.09]	1.04	[0.97–1.11]
*Cat*	1.07	[0.99–1.15]	1.01	[0.93–1.09]	1.00	[0.93–1.08]	1.03	[0.95–1.12]
*Dog*	1.12**	[1.05–1.21]	1.00	[0.93–1.08]	1.00	[0.92–1.08]	1.01	[0.94–1.10]
*Bird*	1.14*	[1.02–1.27]	1.05	[0.93–1.18]	1.03	[0.91–1.17]	1.05	[0.93–1.19]
*Fish*	0.91	[0.77–1.08]	0.96	[0.82–1.14]	0.98	[0.83–1.15]	1.00	[0.84–1.19]

The *N* and Events of each regression depends on the missing values. *, **denote the significance level at 5% and 1%, respectively. Model 1 adjusted for age, gender. Model 2 adjusted for factors in Model 1 $+$ education, financial struggle, country, asthma (except for the fish model), depression. Model 3 adjusted for factors in Model 2 $+$ self-perceived health, no. conditions, no. symptoms (+ moderate physical activity, disability for dog only). Model 4 adjusted for factors in Model 3 $+$ household size, marital status, employment status, nursing home, cohort effects, smoking, alcohol consumption, moderate and vigorous physical activity, and BMI

**Fig. 2:**
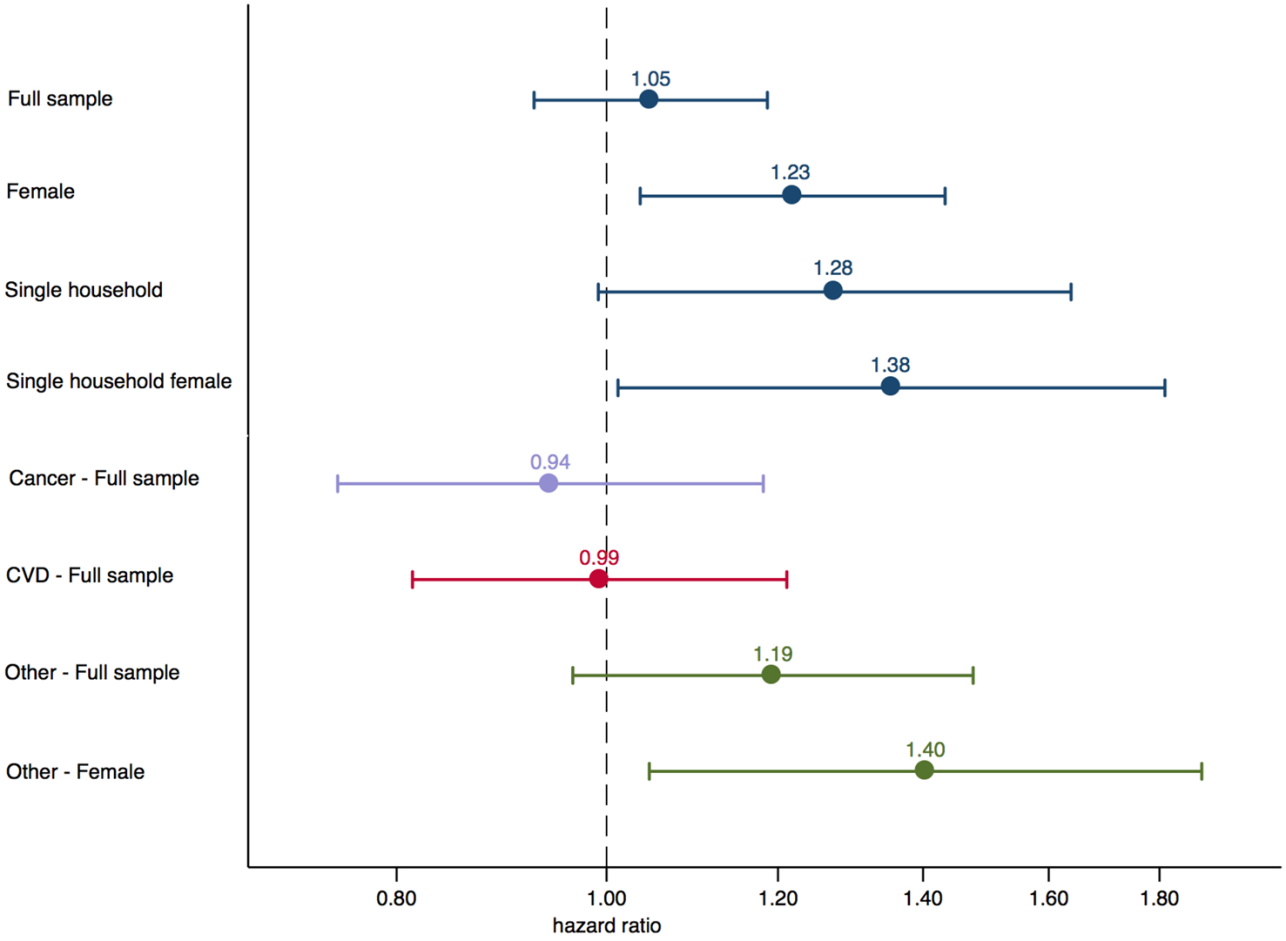
Forest plot for Bird exposure. Estimates are from the fully adjusted model. All-cause mortality is shown in blue, cancer in lavender, CVD in red and Other in green. The graph was partially produced using the -coefplot- command (Jann 2014)

Stratified analyses were carried using the fully adjusted model. The only significant association from the gender stratification was that bird exposure in women increased the rate of death by 23%. In this particular case, models 1 through 3 were also examined (results not shown). The basic model yielded the highest magnitude $[\text {HR}=1.30$ (1.12–1.51)], while the same hazard ratio was estimated from Model 2 and 3 $[\text {HR}=1.19$ (1.02–1.40)]. Bird exposure was also associated with an additional 27% increase in the rate of death for single household owners ($p\, \text {value} =0.066$), albeit not significant in models 1 through 3. To further test the hazard of bird exposure, we analysed data from women who live in single-person households ($n=2964$, Events = 899) using the fully adjusted model. This yielded a significant at the 5%, 38% [95% CI (1.02–1.85)] higher death rate for those bird owners (estimate shown in Fig. 2). Stratified results are presented in Table 4.

**Table 4:** Stratified all-cause mortality hazard ratios and 95% confidence intervals by gender and household size

	Gender				Household			
	Male		Female		Single		Multiple	
	HR	CI	HR	CI	HR	CI	HR	CI
*Any pet*	1.03	[0.94–1.12]	1.06	[0.96–1.16]	1.05	[0.92–1.20]	1.02	[0.95–1.11]
*Cat*	1.03	[0.92–1.14]	1.04	[0.93–1.16]	1.01	[0.86–1.19]	1.02	[0.93–1.12]
*Dog*	1.00	[0.89–1.11]	1.04	[0.93–1.17]	0.96	[0.81–1.14]	1.02	[0.93–1.12]
*Bird*	0.93	[0.79–1.10]	1.23*	[1.04–1.44]	1.28	[0.98–1.66]	1.00	[0.87–1.15]
*Fish*	0.97	[0.79–1.20]	1.08	[0.83–1.40]	1.02	[0.65–1.60]	1.00	[0.83–1.20]

The estimates were obtained using the fully adjusted model (Model 4). Stratified regressions for any pet had: For males *N* = 10,213, Events = 2583; For females *N* = 11,901, Events = 2229; For single-person households *N* = 4200, Events=1335; For multiple-person households *N* = 17,914, Events = 3,477. *, **denote the significance level at 5% and 1%, respectively

### Cancer and CVD mortality

The cause-specific models for cancer, CVD and Other mortality, revealed only a significant, at the 10% level, positive association between bird owners and Other mortality [$\text {HR}=1.19$ (0.97–1.48)]. As we had evidence of effect modification by gender, we stratified the model and found that women bird owners are associated with a significant, at the 5% level, increased rate of mortality of other causes $[\text {HR}=1.40$ (1.05–1.88)] (estimates shown in Fig. 2), but not cancer mortality for women owners of cats or birds. Dog or cat ownership was not associated with CVD mortality, in the full and stratified by CVD diagnosis samples, as previous research had shown (Mubanga et al. 2019; Ogechi et al. 2016), although some vague evidence of a prophylactic relation was there for individuals diagnosed with CVD at baseline $[\text {HR}=0.83$ (0.66–1.04)] (results not shown). Cause-specific estimates are shown in Table 5.

**Table 5:** Cause-specific hazard ratios and 95% confidence intervals for pet exposures from the fully adjusted model

	Cancer		CVD		Other	
	csHR	CI	csHR	CI	csHR	CI
*Any pet*	1.05	[0.93–1.18]	1.00	[0.90–1.12]	0.99	[0.88–1.12]
*Cat*	1.00	[0.86–1.15]	1.08	[0.95–1.23]	0.96	[0.83–1.12]
*Dog*	1.09	[0.95–1.25]	0.94	[0.82–1.07]	0.95	[0.82–1.11]
*Bird*	0.94	[0.75–1.18]	0.99	[0.81–1.21]	1.19	[0.97–1.48]
*Fish*	1.13	[0.86–1.49]	0.87	[0.63–1.20]	0.88	[0.63–1.23]

For the cause-specific model, we use the fully adjusted model and control additionally for cancer or CVD diagnosis at baseline in the respective model. *, **denote the significance level at 5% and 1%, respectively

## Discussion

### Findings in the light of the literature

Overall pet ownership was not associated with survival in this sample of older European adults. In the disaggregated analysis, cats, dogs and fish did not show any significant associations when we controlled for potential confounders other than age and gender. This was true for cancer, CVD and death from other causes as well. For cats and dogs, the findings are in accordance with previous research controlling for sufficient confounders (Ding et al. 2018; Qureshi et al. 2009; Torske et al. 2017; Gillum and Obisesan 2010). For fish, no previous research exists, to the best of our knowledge, and we did not find something that would spark interest in the research community.

The negative associations of bird ownership with the survival of women and/or persons living on their own steers the conversation from lung cancer incidence to perhaps multiple or other causes of death. Although we did not have cancer-specific data to examine the bird-lung cancer relation, our data showed no association with overall cancer mortality in contrast to Buck et al. (2020). But a significant association was there for causes other than cancer and CVD. This category, among other causes, includes respiratory diseases such as psittacosis, allergic alveolitis and asthma, diseases long connected with bird exposure (Gorman et al. 2009).

### Potential mechanisms

The mechanism behind the consistent findings of increased susceptibility of *only* women bird owners in survival studies remains elusive (Adhikari et al. 2019; Buck et al. 2020). Earlier lung-cancer case–control, hospital-based studies that reported adverse effects did include males in their sample (Alavanja et al. 1996; Gardiner et al. 1992; Holst et al. 1988; Kohlmeier et al. 1992; Modigh et al. 1996; Morabia et al. 1998) whereas bird breeder’s lung (allergic alveolitis) probably took the name from male bird breeders. The fact that we had an amplification effect for single-person households is perhaps evidence of a dose–response relationship, in the sense that single-person households are expected to be smaller in size, hence the air volume and change rate (flow) may be more limited. This signifies increased exposure to allergens and dust particulates such as feather dander, as well as fomites, excreta, and mould (Holst et al. 1988). Chronic inhalation of this bioaerosol might be the main biological mechanism behind pathogenesis (Holst 1991).

Interestingly, increased pulmonary vulnerability to environmental exposures (air pollution, as well as smoking) in women has been reported (Tam and Sin 2013). This vulnerability, combined with the aforementioned ‘pulmonary’ hypothesis, could provide a possible explanation for the modification effect by gender among bird owners observed in this study. Details of the bird type, number, and location inside the household will be crucial to elucidate the matter in an observational setting.

### Missingness and censoring

Pet exposures had less than 0.01% missing values. The variables with the most missing values had less than 2% gaps and concerned education, financial struggle at the household, and BMI. The sample consisted of 22,114 complete cases that represent the 95.02%. A dataset with less than 5% missing values total, is not expected to produce biased estimates in a complete case analysis regardless of the data generating process of the missingness. Nevertheless, pairwise deletion was preferred.

The assumption of independent (right) censoring is crucial for the validity and unbiasedness of the standard TTE analysis results. In medical research, the health status of a participant is the main determinant for attrition and, consequently, right-censoring. This is a big issue when one is studying the treatment effects since more/less healthy individuals systematically remain in the study. Evidence for informative censoring, although uncommon in longitudinal surveys like SHARE, exists due to the not-at-random data generating process caused by attrition, especially when the follow-up is long.

### Modelling and inferential difficulties

As mentioned earlier in the paper, identifying confounders is not easy with pet exposure. The absence of hard confounders, as gradual movements towards the null indicate when adding variables, is not a problem. On the other hand, identifying mediators in order to estimate the direct effect is a difficult task when the beginning of the exposure is unknown, since pets have been linked with all sorts of health benefits, as well as some hazards. It is not clear though if medical covariates also influence weakly the selection of pets and are, therefore, weak confounders.

The present methodology is not an attempt on mediation analysis and does not aim to estimate the indirect effects. The observed change in the hazard ratios from the two Cox PH models (with and without potential mediators) does not have a clear causal interpretation as it would in linear models and techniques that estimate the baseline hazard and deal with violations of the PH assumption would need to be employed for mediation analysis with Cox models (Wang and Albert 2017). Nevertheless, it provides indications for mediating or (weak) confounding effects of the added variables.

### Limitations

Since the questionnaire was not designed to study this particular hypothesis, some limitations regarding the measurement of the exposure are inevitable. The survey does not provide detailed information regarding the human–animal interaction (e.g., the level of attachment to the pet, the duration of the ownership, whether the participant is the actual caretaker), therefore a dose–response effect cannot be estimated. Moreover, the breed is not known, hence further analysis cannot take place. The beginning and end of the exposure are also unknown—a common drawback of observational studies compared to experimental designs. Consequently, the amount pet exposure varies in time is not captured in our data. In simpler terms, given that the life expectancy of humans is much higher than any of the examined pets, it remains unknown if and when another pet was acquired in the case of a pet’s death.

Data from the survey are self-reported. This includes mortality data as well. Both can introduce an amount of measurement error, particularly in cause-specific mortality. Despite the surveys’ design for random sampling and weights provided, our sub-sample does not allow for these weights to be used, nor can we claim that this is a representative sample of older European residents. Despite this limitation in internal validity, the generalisability remains higher than the rest of the literature since it pools individuals from different countries. A case can be made for residual confounding, although frailty models did not show the presence of unobserved heterogeneity even in models severely lesser than the fully adjusted ones.^11^ Nevertheless, rurality and diet are factors the authors wish they could control for.^12^

Since we run multiple models in order to test several hypotheses, an increased risk of type I error exists. However, given the exploratory nature of our analysis, as well as predefined research objectives, no *p* value adjustments were made. Finally, mortality is not a measure of quality of life; therefore, this study cannot infer whether pet owners live a better life as was previously suggested by Hughes et al. (2020).

## Conclusions

No association was evident between pet ownership and survival in this sample of older adults. As similar evidence is accumulating, more detailed cohort data would probably be necessary to challenge and verify the existing literature. Bird ownership, especially among older women, requires further investigation with longitudinal studies that include a more comprehensive measurement of the exposure and outcome. Given the existing knowledge, no public health recommendation can be safely made in terms of survival yet. Nevertheless, bird owners, and especially older women and individuals with lung conditions, or those with significant risk factors for pulmonary pathologies, are advised to air the room of the bird frequently, or alternatively, depending on the availability of space, place the pet outdoors in a safe environment.

Pets can become a faithful companion for older individuals, especially at times when the feelings of isolation and loneliness intensify. Their effect and importance on the lives of humans cannot be captured by a single variable or studied using a single outcome. Nevertheless, future research should focus on obtaining details on the beginning of the exposure and the level of the interaction between pets and humans.

## Data Availability

The datasets used for this analysis are available from the SHARE website, http://www.share-project.org/home0.html.

## Footnotes

1 Studies for established CVD also exist (see for example Chowdhury et al. 2017; Mubanga et al. 2019). See also Kramer et al. (2019) for a review on dog ownership and CVD survival.

2 See Börsch-Supan and Jürges (2005), pp. 28–37 for details on the sampling procedure.

3 Numbers vary depending on the measurement methodology.

4 Countries include: Austria, Germany, Sweden, Netherlands, Spain, Italy, France, Denmark, Greece, Switzerland, Belgium, Israel, Czech Republic, Poland and Ireland.

5 CVD includes deaths from a heart attack, a stroke and other cardiovascular related illness.

6 Other causes include: Respiratory disease, disease of the digestive system, severe infectious disease, accidents, and other not previously mentioned causes.

7 Nursing homes were defined as “...institutions sheltering older persons who need assistance in activities of daily living, in an environment where they can receive nursing case, for short or long stays” and the question was ‘During the last twelve months, have you been in a nursing home overnight?’. This variable could also belong to the medical covariates.

8 A dummy variable was created to indicate single-person households, for stratified analysis.

9 Available responses were the same.

10 Euro-D is a validated scale that ranges from 0 to 12 and it is used to measure geriatric depression. The factors used in the formation of the scale by SHARE include: depression, pessimism, suicidality, guilt, sleep, interest, irritability, appetite, fatigue, concentration, enjoyment and tearfulness. For more details on Euro-D see Prince et al. (1999).

11 Gompertz gamma frailty models were used to perform a likelihood ratio test between the frailty and non-frailty model.

12 The corresponding variable for rurality had too many missing values to even consider multiple or another form of imputation and dietary information were not available.
